# Supplementary material for: Simultaneously Enhancing the Strength, Plasticity, and Conductivity of Copper Matrix Composites with Graphene-Coated Submicron Spherical Copper
Source: Nanomaterials (Basel). 2022 Mar 21;12(6):1025. doi: 10.3390/nano12061025 (PMC8951116; doi:10.3390/nano12061025)
Supplement: Supplementary file 1 [file nanomaterials-12-01025-s001.zip › nanomaterials-1629288-supplementary.pdf]

*Supporting Information*

# Simultaneously Enhancing the Strength, Plasticity, and Conductivity of Copper Matrix Composites with Graphene-Coated Submicron Spherical Copper

**Yulong Yang [1,2,3], Yilong Liang [1,2,3,*], Guanyu He [1,2,3], and Pingxi Luo [1,2,3]**

[1] College of Materials and Metallurgy, Guizhou University, Guiyang 550025, China; mryyl252@163.com (Y.Y.);hgy2726220@163.com (G.H.); lpx15186160957@163.com (P.L.)
[2] Key Laboratory for Mechanical Behavior and Microstructure of Materials of Guizhou Province, Guiyang 550025, China
[3] National & Local Joint Engineering Laboratory for High-performance Metal Structure Material and Advanced Manufacturing Technology, Guiyang 550025, China
* Correspondence: ylliang@gzu.edu.cn

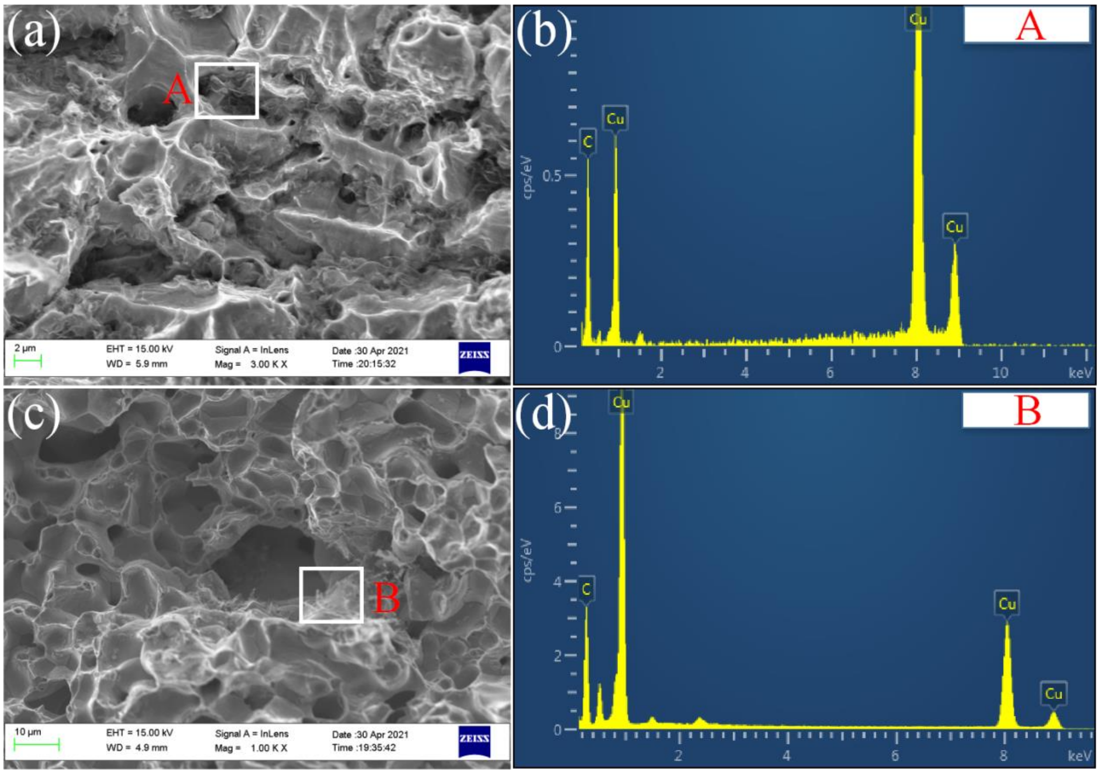


**Figure S1.** (**a**) Fracture SEM images of SSCu@rGO/Cu composites, (**b**) the EDS image of the white box at position A in Subfigure (**a**), (**c**) fracture SEM images of rGO/Cu composites, and (**d**) the EDS image of the white box at position B in Subfigure (**c**).

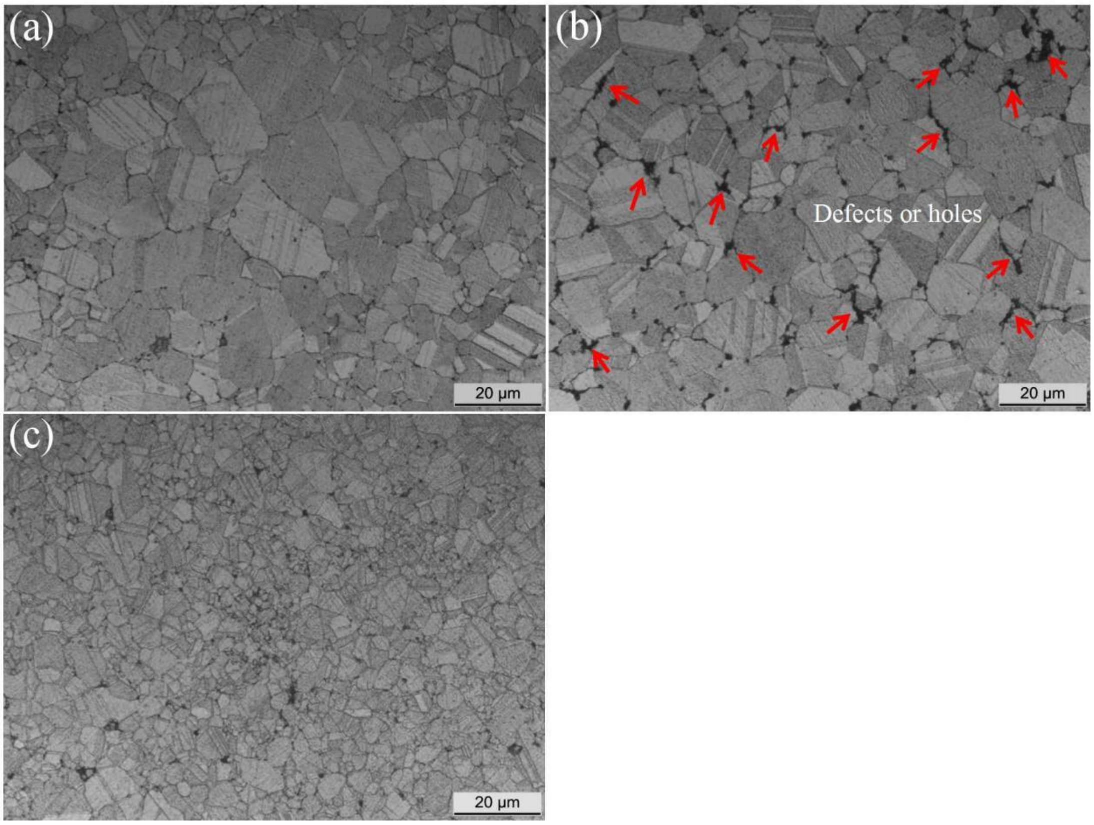


**Figure S2.** (**a**) OM image of pure Cu surface, (**b**) OM image of rGO/Cu composites surface, and (**c**) OM image of SSCu@rGO/Cu composites surface.

**Table S1.** Hardness measurement value.

| Samples | Handness (HV) | | | | |
|---|---|---|---|---|---|
| Pure Cu | 81.8 | 83.7 | 83.3 | 83.5 | 85.7 |
| 0.1%rGO/Cu | 85.9 | 85.1 | 85.7 | 87.2 | 89.8 |
| 0.3%rGO/Cu | 60.2 | 69.3 | 65.6 | 59.5 | 59.3 |
| 0.5%rGO/Cu | 46.0 | 47.8 | 47.8 | 49.2 | 47.6 |
| SSCu/Cu | 83.8 | 90.9 | 84.8 | 90.9 | 90.6 |
| SSCu@0.1%rGO/Cu | 106.0 | 109.9 | 105.2 | 109.5 | 103.0 |
| SSCu@0.3%rGO/Cu | 109.1 | 101.5 | 104.5 | 114.1 | 104.8 |
| SSCu@0.5%rGO/Cu | 97.3 | 95.0 | 96.0 | 92.5 | 94.0 |
